# Effectiveness of lenvatinib plus immune checkpoint inhibitors in primary advanced hepatocellular carcinoma beyond oligometastasis

**DOI:** 10.1002/ctm2.1214

**Published:** 2023-02-28

**Authors:** Xiao‐Hui Wang, Chang‐Jun Liu, Hao‐Quan Wen, Xiao‐Hui Duan, Yu‐Qing Jiao, Yu‐Jiang Liu, Min‐Shan Chen, Kang‐Shun Zhu, Xian‐Hai Mao, Qun‐Fang Zhou

**Affiliations:** ^1^ Department of Hepatobiliary Surgery Hunan Provincial People's Hospital, The First Affiliated Hospital of Hunan Normal University, Changsha Hunan Province China; ^2^ Department of Minimally Invasive Interventional Radiology The Second Affiliated Hospital of Guangzhou Medical University Guangzhou China; ^3^ Department of Radiology The Second Affiliated Hospital of Guangzhou Medical University Guangzhou China; ^4^ Department of Interventional Ultrasound Chinese PLA General Hospital Beijing China; ^5^ Department of Liver Surgery Sun Yat‐Sen University Cancer Center Guangzhou Guangdong China

**Keywords:** advanced hepatocellular carcinoma, beyond oligometastasis, lenvatinib, prognosis, programmed death receptor‐1 inhibitor

## Abstract

**Background:**

Targeted therapy combined with immune checkpoint inhibitors is considered a promising treatment for primary advanced hepatocellular carcinoma (HCC). Nevertheless, the difference between synchronous and asynchronous treatment of lenvatinib with programmed death receptor‐1 (PD‐1) inhibitor in advanced HCC is still unclear. The aim of this investigation is to evaluate the effectiveness of synchronous and asynchronous of lenvatinib and PD‐1 inhibitor on the advanced HCC beyond oligometastasis.

**Methods:**

In this study, 213 patients from four institutions in China were involved. Patients were split into two collections: (1) lenvatinib plus PD‐1 inhibitor were used synchronously (synchronous treatment group); (2) patients in asynchronous treatment group received PD‐1 inhibitor after 3 months of lenvatinib treatment prior to tumour progression. To analyse progression‐free survival (PFS), overall survival (OS), efficacy and safety of patients in both groups, we employed propensity score matching (PSM).

**Results:**

The 6‐, 12‐ and 24‐month OS rates were 100%, 93.4% and 58.1% in the synchronous treatment group and 100%, 71.5% and 25.3% in the asynchronous treatment group, respectively. In contrast to the asynchronous treatment group, the group treated synchronously exhibited a substantially enhanced OS (hazard ratio [HR], 0.45; 95% confidence interval [CI], 0.30–0.66; *p* < .001). The 6‐, 12‐ and 18‐month PFS rates were 82.6%, 42.6% and 10.8% in the synchronous treatment group and 63.3%, 14.2% and 0% in the asynchronous treatment group, respectively. A significant difference was observed in the PFS rate (HR, 0.46; 95% CI, 0.33–0.63; *p* < .001) between the two collections.

**Conclusions:**

Patients with advanced HCC beyond oligometastasis, simultaneous administration of lenvatinib and PD‐1 inhibitor led to significant improvements in survival.

## Graphical abstract

The treatment of advanced hepatocellular carcinoma still faces many challenges.

Lenvatinib combined with programmed death receptor‐1 (PD‐1) inhibitor is a promising treatment for advanced hepatocellular carcinoma.

Synchronous combination of lenvatinib with PD‐1 inhibitor resulted in significant survival improvements in patients with advanced hepatocellular carcinoma.

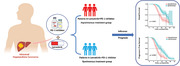

## 1 INTRODUCTION

Early‐phase hepatocellular carcinoma (HCC) is confined to liver and has been recommended for resection or ablation.^1^ Unfortunately, many patients present or subsequently develop distant metastases, which are generally regarded as advanced stage.^2^ The mainstay therapy for metastatic HCC is typically systemic therapy.^3^ Evidence showed that recurrence and extrahepatic metastasis have been the main causes of tumour‐related death.^4^ The coincidence of HCC with metastasis is relatively high.^5^ In all extrahepatic metastases, the most common metastatic organ is the lung, followed by bones, lymph nodes and adrenal glands, and metastases are often associated with comparatively high tumour burden and vascular invasion.^6^, ^7^ However, some patients with extrahepatic metastases and vascular invasion may still not be regarded as terminal stage if they have good liver function and physical state.^8^ With the development of new treatments, the prognosis of patients with multiple metastases has improved.^9^ Although treatments are relatively limited, heterogeneity still exists in this population due to differences in tumour characteristics, tumour burden, organs involved and the degree of liver dysfunction.^10^

The definition of oligometastasis was a patient with five or fewer metastases that were potentially amenable to local approaches.^11^, ^12^ Compelling studies have proven that local therapy could obtain the long‐term disease‐free survival after killing the foci within oligometastasis.^13^, ^14^, ^15^ However, for the patients beyond oligometastasis, the prognosis was clearly worse than those within oligometastasis.^16^ Current systemic therapies have been exhibited to be increasingly effective and tolerable options for controlling advanced HCC beyond oligometastasis.^17^

Currently, derived from the latest Barcelona Clinical Liver Cancer (BCLC) treatment algorithm recommendation, atezolizumab combined with bevacizumab is the first‐choice treatment for advanced HCC.^18^ In addition, lenvatinib also holds a significant part in the management of advanced HCC.^19^ Despite advancements in relieving of advanced HCC, there remains a need for innovative combination therapies to overcome the limitations of monotherapy.^20^ Immunotherapies, including immune checkpoint inhibitors, such as programmed death receptor‐1 (PD‐1) inhibitor, have revealed promising results in advanced HCC.^21^, ^22^ The combination of PD‐1 inhibitor with lenvatinib has shown more potent antitumour effects in clinical trials and is now the hotspot in clinical application.^23^, ^24^, ^25^, ^26^ The rationale for this combination is based on that lenvatinib could inhibit neovascularization and immunosuppressive effects of tumor microenvironments, and such inhibition would improve the clinical benefit of PD‐1 antibodies by boosting the antitumor immune response.^25^ In clinical application, some patients received the lenvatinib and PD‐1 inhibitor at the same time, and some patients received the PD‐1 inhibitor after unsatisfactory lenvatinib alone.^27^ However, there has been no research reporting the difference in synchronous or asynchronous of lenvatinib and PD‐1 inhibitor in advanced HCC. Therefore, in the retrospective multi‐centre study, we intend to analyse the effectiveness of synchronous and asynchronous of lenvatinib and PD‐1 inhibitor on the advanced HCC beyond oligometastasis.

## 2 MATERIALS AND METHODS

### 2.1 Patients and study design

**FIGURE 1:**
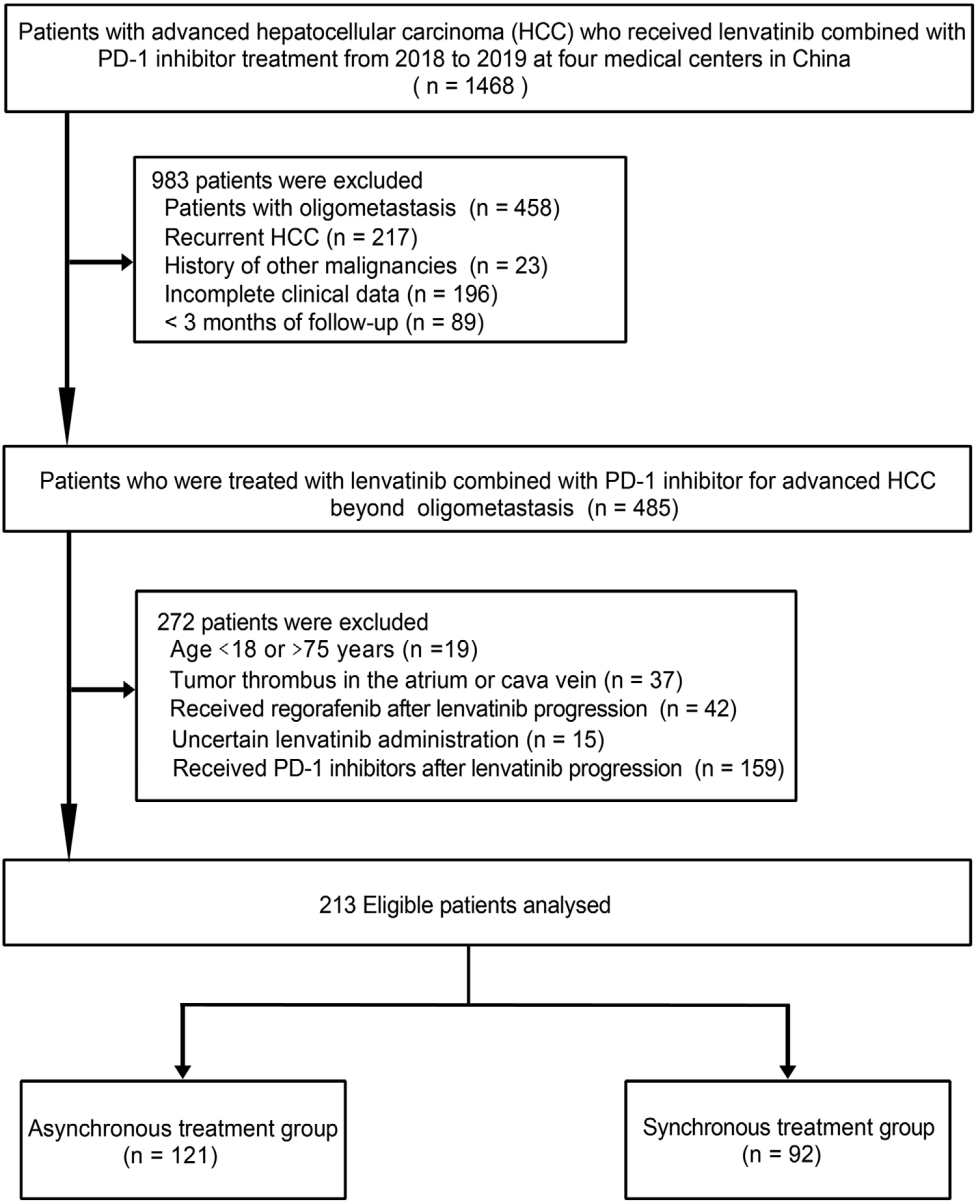
Flow chart of patient selection. PD‐1, programmed death receptor‐1.

The research involved patients who had advanced HCC beyond oligometastasis from January 2018 to December 2019 at the Second Affiliated Hospital of Guangzhou Medical University, Chinese PLA General Hospital, Sun Yat‐Sen University Cancer Center, Hunan Provincial People's Hospital. Individuals fulfilling the following criteria were enrolled: (1) primary unresectable HCC confirmed by clinical or histopathology; (2) BCLC stage C, beyond oligometastasis (more than five metastases)^28^; (3) Child–Pugh class A or B; (4) no prior history of other malignancies; (5) no tumour thrombus in the atrium or vena cava. Individuals who met the exclusion criteria were not eligible for this investigation: (1) recurrent HCC; (2) under 18 or over 75 years; (3) advanced HCC with five or fewer metastases; (4) incomplete clinical data; (5) patients who could not be reached or tracked after 3 months of treatment initiation; (6) patients who received regorafenib after lenvatinib progression; and (7) patients who were treated with PD‐1 inhibitors after lenvatinib progression. Patient selection is presented in Figure 1.

### 2.2 Treatment and assessment of response

A contrast‐enhanced computed tomography (CT) or magnetic resonance imaging (MRI) was evaluated in all patients 2 weeks prior to receiving lenvatinib treatment. These patient cohorts were categorised into two groups. (1) Lenvatinib plus PD‐1 inhibitor were used synchronously (synchronous treatment group); this group of participants was treated with lenvatinib and PD‐1 inhibitor at the same time, or patients who received PD‐1 inhibitor within 1 week of lenvatinib were also included in this group. (2) Without evidence of tumour progression (TP), patients treated with PD‐1 inhibitors after more than 3 months of lenvatinib alone (at least two or more imaging assessments have been performed) (asynchronous treatment group).

Details on treatment initiation, completion and adverse events (AEs) were methodically recorded. Eight milligrams per day of lenvatinib was recommended for patients weighing less than 60 kg, and those weighing 60 kg or more were treated with 12 mg per day. The medication was taken orally once a day. As per the lenvatinib administration guidelines, treatment was either suspended or the drug dose altered in patients who experienced severe AEs of grade 3 or above or any grade 2 drug‐related AEs that were deemed unacceptable. In such cases, alternative treatment was recommended upon TP. For PD‐1 inhibitor (including pembrolizumab, nivolumab, sintilimab, toripalimab, camrelizumab), the dose was applied according to the drug instructions. Patients received imaging examination (MRI or CT) by using triphasic scanning technique. Tumour evaluations were performed every 6 weeks (regardless of dose interruption) until radiological progression. Patients without disease progression continued the evaluation every 6 weeks.

### 2.3 Outcomes and definitions

The main objectives of this investigation were to assess overall survival (OS). Progression‐free survival (PFS), efficacy and safety were included as the secondary endpoints. The duration of OS was computed between the initiation of lenvatinib treatment and death or the final follow‐up visit. Meanwhile, PFS was determined between the start of lenvatinib treatment and either the appearance of TP or the date of the final follow‐up. Tumour staging was determined through systemic imaging (positron emission tomography/CT or contrast‐enhanced MRI or CT of abdomen or brain, contrast‐enhanced CT of the chest or bone scan). Liver function was measured by the albumin–bilirubin (ALBI) grade.^29^ Portal vein tumour thrombus (PVTT) was divided into four categories based on Cheng's criteria.^30^ Type I, tumour thrombus affecting the segmental branches or higher of the portal vein. Type II, tumour thrombus affecting either the right or left portal vein. Type III, tumour thrombus affecting the main portal vein. Type IV, tumour thrombus affecting the superior mesenteric vein. The local therapies received by the patients, for example, ablation, transarterial chemoembolisation (TACE), hepatic arterial infusion chemotherapy (HAIC) and radiotherapy, before starting lenvatinib were recorded. Additionally, drug‐related complications were also documented.

### 2.4 Follow‐up

This investigation's follow‐up period concluded on 30 September 2021. Patients underwent evaluation at minimum intervals of 6 weeks following the initiation of treatment. Each follow‐up visit consisted of image examination (contrast‐enhanced CT/MRI), and experimental assessments involving alanine aminotransferase (ALT), aspartate aminotransferase (AST), albumin, prothrombin time (PT), bilirubin and alpha‐fetoprotein (AFP). Up to two tumours per organ and a maximum of five tumours in total were selected as target tumours. Tumour imaging responses included were evaluated based on the Response Evaluation Criteria in Solid Tumours 1.1 (RECIST 1.1), which include partial response (PR), complete response (CR), progressive disease (PD) and stable disease (SD).^31^ CR was described as a lack of any tumour artery improvement. PR was defined as target tumour diameter reduction ≥30%. PD was identified by either a minimum growth of 20% in the overall size of the tumours being targeted or the emergence of new lesions. SD was identified by not conforming to the principles for CR, PR or PD. The objective response rate (ORR) was the sum of CR and PR, while the disease control rate (DCR) was calculated as the combination of CR, PR and SD.

### 2.5 Statistical analysis

In order to minimise alternative factors and sampling bias, propensity score‐matching (PSM) study was involved. PSM was accomplished by equating the two groups based on 18 variables, including age, sex, maximum tumour size, cirrhosis, portal hypertension, location of metastasis, history of local treatment, number of tumours in the liver, platelet count, haemoglobin, creatinine, hepatitis B surface antigen (HBsAg), AFP, AST, ALT, PT, PVTT and ALBI grade. For propensity score matching, a nearest‐neighbor 1:1 matching scheme with a caliper size of 0.2 was used (as shown in Figure S1). The matched cohort was utilised to test the OS and PFS in the two collections through the application of a log‐rank test.

For comparison of continuous variables, the independent *t*‐test was employed, while categorical variables were investigated through the chi‐square test. The OS and PFS rates were investigated by using the Kaplan–Meier method for survival analysis, with group comparisons utilising the log‐rank test. To investigate the impact of potential prognostic factors on clinical outcomes, both univariate and multivariable Cox proportional hazards models were employed. All statistical analyses were performed using both R software for Windows (version 3.6.4; http://www.r‐project.org) and the Statistical Package for the Social Science software (version 22.0; SPSS Inc., Chicago, IL, USA) on a Windows platform. *p*‐Value <.05 was used as the criteria for determining statistical significance.

## 3 RESULTS

### 3.1 Baseline characteristics

In the analysis, 213 patients had a median age of 51 years and a range of 25–75 years. Of these patients, 185 (86.9%) were male. There were 121 (56.8%) patients who received the PD‐1 inhibitor after 3 months of lenvatinib treatment prior to TP (asynchronous treatment group); a total of 92 (43.2%) patients received lenvatinib plus PD‐1 inhibitor at the same time (synchronous treatment group). There were 106 (49.8%) patients with different PVTT involvement and 180 (84.5%) patients had a history of local treatment before lenvatinib. The PSM analysis resulted in two balanced patient cohorts, each with 85 patients, in the synchronous and asynchronous treatment groups. The baseline variables were well matched with a standardised mean deviation of no more than 10% for all variables analysed, as demonstrated in Figure S2. A summary of the patients’ demographic and clinicopathological features is provided in Table 1.

**TABLE 1:** Baseline characteristics of patients with advanced hepatocellular carcinoma beyond oligometastasis in different treatment groups.

	Entire cohort			Propensity score‐matched cohort (1:1 ratio)		
Characteristics	Asynchronous group (*n* = 121)	Synchronous group (*n* = 92)	*p‐*Value	Asynchronous group (*n* = 85)	Synchronous group (*n* = 85)	*p‐*Value
Age^a^ (years)	51.0 (28–75)	50.0 (25–75)	.615	51.0 (30–75)	51.0 (25–75)	.994
Sex (male), *n* (%)	106 (87.6)	79 (85.9)	.711	74 (87.1)	73 (85.9)	.823
HBsAg positive, *n* (%)	107 (88.4)	80 (87.0)	.745	76 (89.4)	75 (88.2)	.808
Haemoglobin^a^ (g/dL)	13.3 (7.4–18.1)	13.7 (6.8–18.3)	.283	13.5 (7.4–18.1)	13.6 (6.8–18.3)	.929
Platelet^a^ (10^9^/L)	191 (70–573)	189 (75–455)	.416	193 (70–509)	190 (76–455)	.903
ALT^a^ (U/L)	42.2 (4.4–522.6)	39.0 (8.7–502.2)	.128	38.2 (6.0–522.6)	38.0 (8.7–502.2)	.508
AST^a^ (U/L)	58.1 (7.5–619.8)	53.0 (9.1–603.0)	.039	54.9 (7.5–491.0)	53.2 (9.1–501.2)	.549
PT^a^ (s)	13.5 (12.5–18.0)	13.1 (11.8–18.7)	.217	13.2 (12.5–18.0)	13.0 (11.8–18.3)	.761
CRE^a^ (μmol/L)	68.8 (31.7–299.6)	67.5 (29.0–201.0)	.520	67.8 (31.7–277.0)	68.0 (29.0–200.5)	.430
AFP (ng/mL)			.421			.715
≤20 ng/mL	27 (22.3)	20 (21.7)		14 (16.5)	18 (21.2)	
20–400 ng/mL	38 (31.4)	22 (23.9)		22 (25.9)	22 (25.9)	
>400 ng/mL	56 (46.3)	50 (54.4)		49 (57.6)	45 (52.9)	
Maximum tumour size^a^ (cm)	7.9 (4.2–20.0)	8.7 (4.9–18.8)	.401	8.1 (4.2–20.0)	8.5 (4.9–18.8)	.715
Tumour number in liver, *n* (%)			.594			.607
≤3	29(24.0)	25 (27.2)		22 (25.9)	25 (29.4)	
>3	92 (76.0)	67 (78.8)		63 (74.1)	60 (70.6)	
History of local treatment			.632			.834
No	20 (16.5)	13 (14.1)		14 (16.5)	13 (15.3)	
Yes	101 (83.5)	79 (85.9)		71 (83.5)	72 (84.7)	
Metastasis location, *n* (%)			.455			.858
Lung	49 (40.5)	42 (45.7)		37 (43.5)	38 (44.7)	
Other organs	38 (31.4)	31 (33.7)		26 (30.6)	28 (32.9)	
Lung + other organs	34 (28.1)	19 (20.6)		22 (25.9)	19 (22.4)	
Cirrhosis (yes), *n* (%)	81 (66.9)	60 (65.2)	.792	57 (67.1)	56 (65.9)	.871
Portal hypertension, *n* (%)			.687			.873
No	73 (60.3)	58 (63.0)		54 (63.5)	55 (64.7)	
Yes	48 (39.7)	34 (37.0)		31 (36.5)	30 (35.3)	
PVTT, *n* (%)			.105			.962
No	55 (45.4)	52 (56.6)		49 (57.6)	50 (58.8)	
I	6 (5.0)	7 (7.6)		6 (7.1)	4 (4.7)	
II	23 (19.0)	13 (14.1)		14 (16.5)	13 (15.3)	
III	31 (25.6)	13 (14.1)		11 (12.9)	13 (15.3)	
IV	6 (5.0)	7 (7.6)		5 (5.9)	5 (5.9)	
ALBI grade, *n* (%)			.570			1.000
Grade 1	50 (41.3)	44 (47.8)		38 (44.7)	38 (44.7)	
Grade 2	66 (54.1)	43 (46.8)		44 (51.8)	44 (51.8)	
Grade 3	5 (4.1)	4 (4.4)		3 (3.5)	3 (3.5)	

*Note*: Data are *n* (%) and ranges.

Abbreviations: AFP, alpha‐fetoprotein; ALBI, albumin–bilirubin; ALT, alanine aminotransferase; AST, aspartate aminotransferase; CRE, creatinine; HBsAg, hepatitis B surface antigen; PT, prothrombin time; PVTT, portal vein tumour thrombus.

^a^ Presented as median (range).

### 3.2 Effect of treatment patterns on overall survival

**FIGURE 2:**
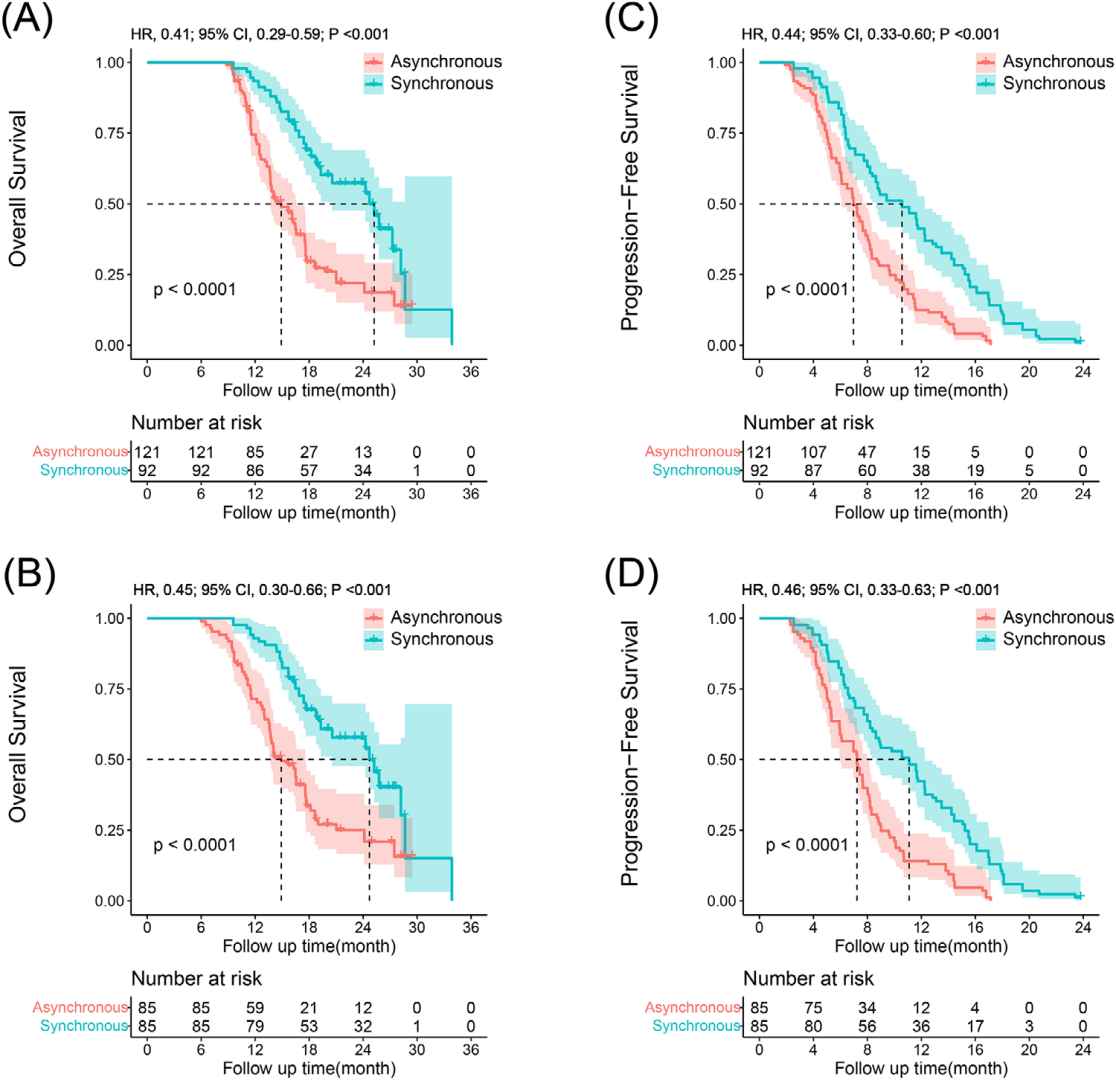
Kaplan–Meier curves of overall survival in the entire cohort (A) and in the propensity score‐matched cohort (B), and progression‐free survival in the entire cohort (C) and in the propensity score‐matched cohort (D) of patients with advanced hepatocellular carcinoma (HCC) beyond oligometastasis who were treated with lenvatinib plus programmed death receptor‐1 (PD‐1) inhibitor. CI, confidence interval; HR, hazard ratio.

Out of the entire cohort, 138 (64.8%) patients died, with 88 (63.8%) deaths occurring in the asynchronous treatment collection and 50 (36.2%) in the synchronous treatment collection. The OS rates were recorded as 100% after 6 months, 82.7% after 12 months and 38.2% after 24 months. Before propensity matching, the 6‐, 12‐ and 24‐month OS rates in the synchronous treatment group were recorded as 100%, 93.4% and 56.9%, respectively. Among asynchronous treatment group, the related OS rates were 100%, 74.6% and 22.2% (Figure 2A). After propensity matching, the 6‐, 12‐ and 24‐month OS rates were 100%, 93.4% and 58.1% in synchronous treatment group and 100%, 71.5% and 25.3% in asynchronous treatment group, respectively (Figure 2B). According to the results, it exhibited that the synchronous treatment group had a substantial improvement in OS compared to the asynchronous treatment, throughout the whole group (hazard ratio [HR], 0.41; 95% confidence interval [CI], 0.29–0.59; *p* < .001) and in the PSM cohort (HR, 0.45; 95% CI, 0.30–0.66; *p* < .001).

**FIGURE 3:**
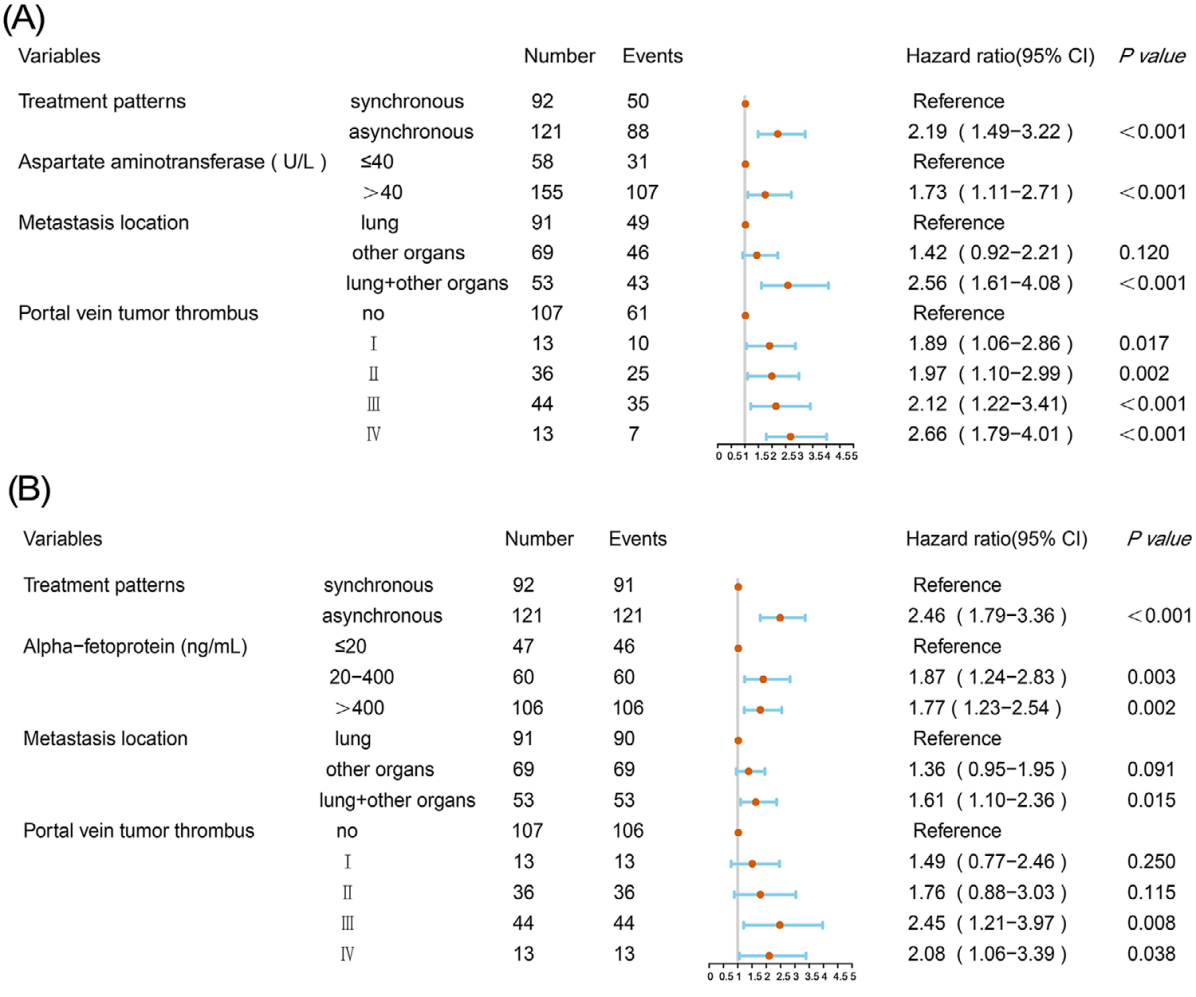
Multivariate analysis and forest plot of the hazard ratio of overall survival (A) and progression‐free survival (B) in the entire cohort. CI, confidence interval.

Multivariate analysis exhibited that treatment patterns, metastasis location, AST level and PVTT were significant factors contributing to mortality in patients with advanced HCC beyond oligometastasis (Figure 3A). Results from the univariate examination of OS and PFS are summarised in Table S1.

### 3.3 Effect of treatment patterns on progression‐free survival

Throughout the whole group, the median PFS were 10.5 and 6.9 months in synchronous and asynchronous treatment groups, respectively. Before propensity matching, the evaluated PFS rates for the 6‐, 12‐ and 18‐month intervals were 83.7%, 41.5% and 11.8% in synchronous treatment group and 64.0%, 12.6% and 0% in asynchronous treatment group, respectively (Figure 2C). Following propensity matching, the 6‐, 12‐ and 18‐month PFS rates were 82.6%, 42.6% and 10.8% in synchronous treatment group and 63.3%, 14.2% and 0% in asynchronous treatment group, respectively (Figure 2D). The results exhibited that the synchronous treatment group had a meaningfully improved PFS compared to asynchronous treatment group, throughout the overall group (HR, 0.44; 95% CI, 0.33–0.60; *p* < .001) and in the PSM cohort (HR, 0.46; 95% CI, 0.33–0.63; *p* < .001).

Multivariate analysis results showed that treatment patterns, metastasis location, AFP level and PVTT were significant factors that influenced TP in patients with advanced HCC beyond oligometastasis (Figure 3D).

### 3.4 Subgroup analysis of prognosis of metastasis location

**FIGURE 4:**
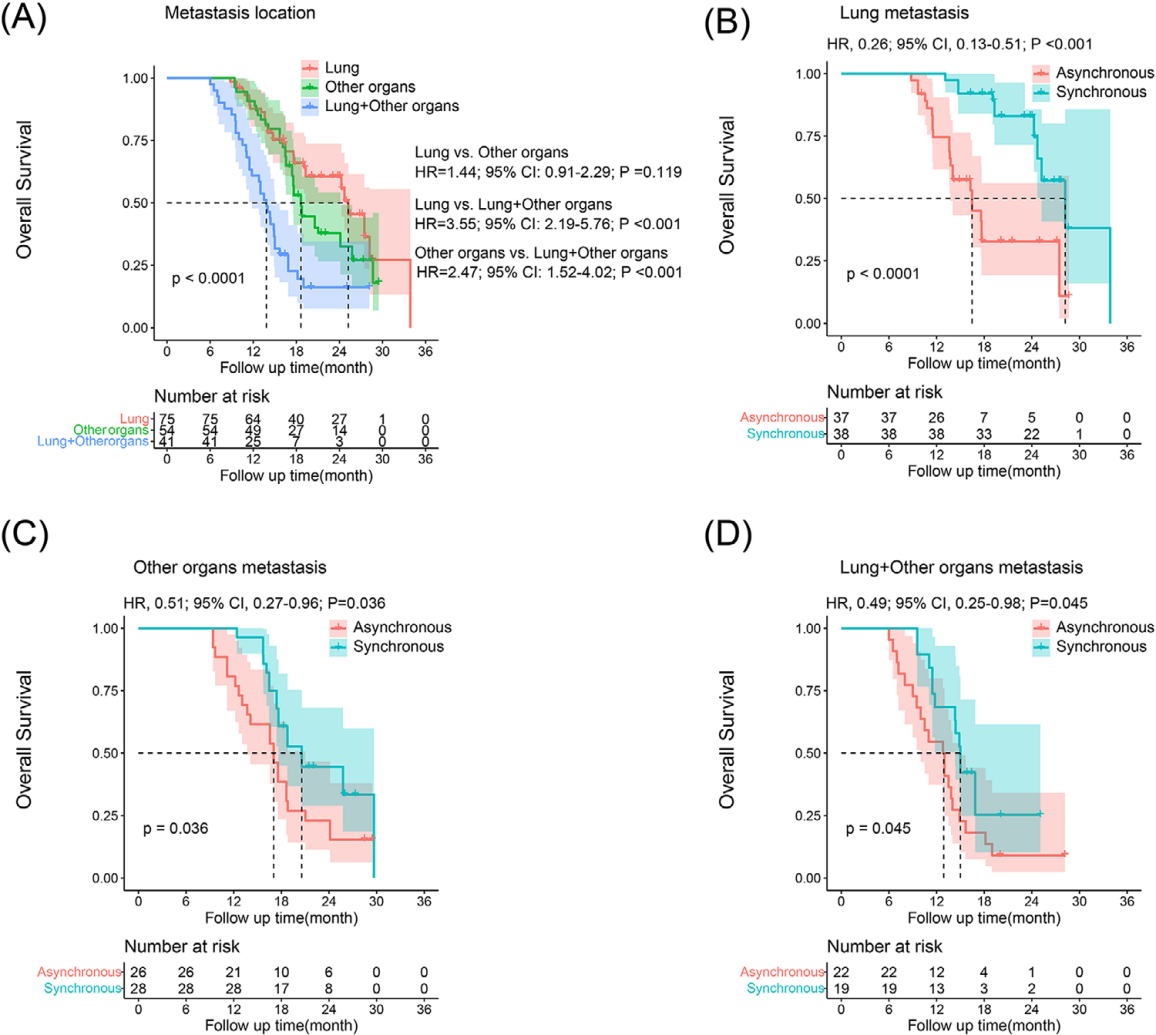
Kaplan–Meier curves for overall survival (OS) in patients with advanced hepatocellular carcinoma (HCC) beyond oligometastasis of different treatment groups. The OS rate of lung metastasis, other organs metastasis and lung plus other organs metastasis in propensity score‐matching (PSM) cohort (A). The OS rate of lung metastasis (B), other organs metastasis (C) and lung plus other organs metastasis (D) in synchronous and asynchronous treatment groups. CI, confidence interval; HR, hazard ratio.

**FIGURE 5:**
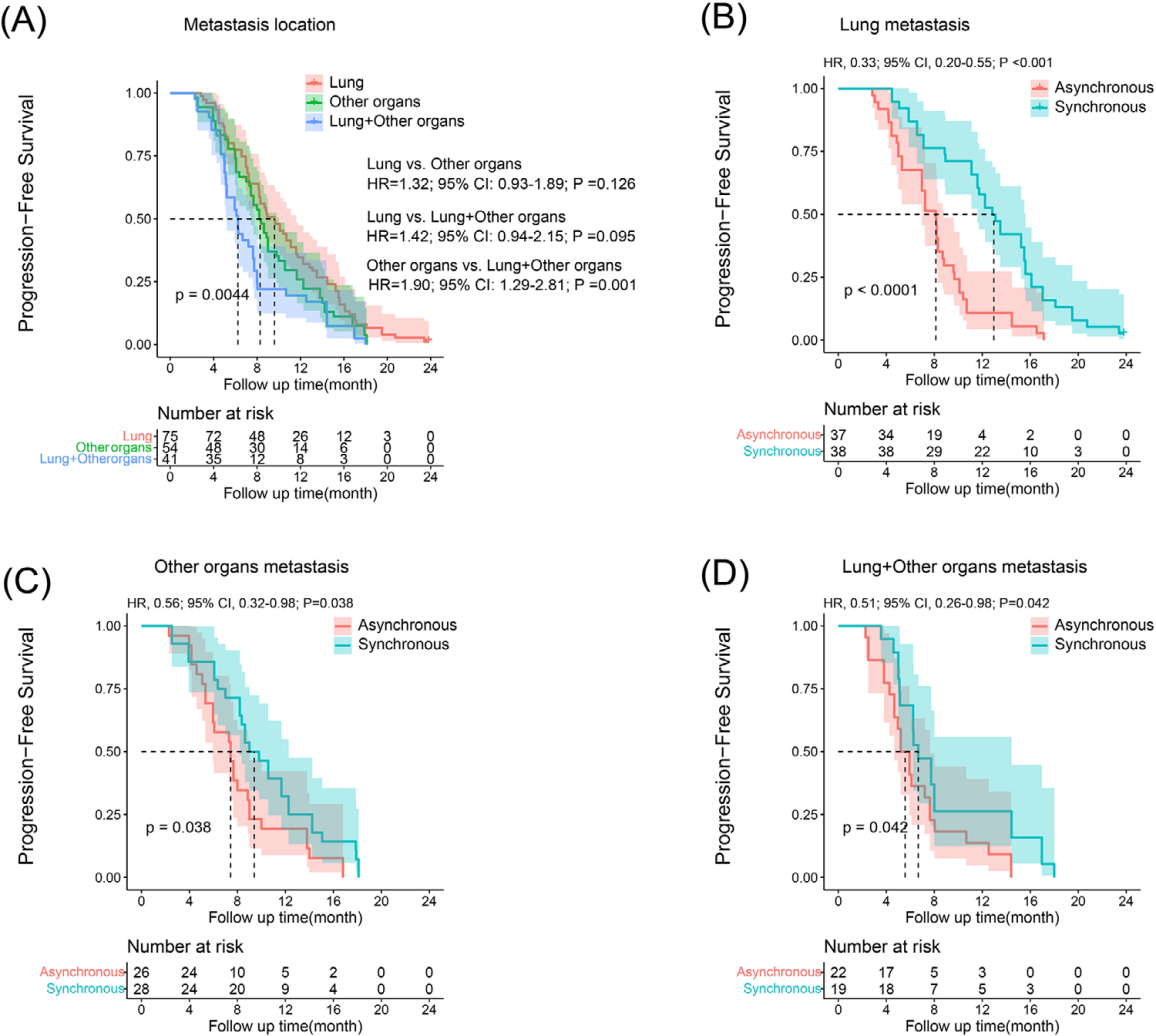
Kaplan–Meier curves for progression‐free survival (PFS) in patients with advanced hepatocellular carcinoma (HCC) beyond oligometastasis of different treatment groups. The PFS rate of lung metastasis, other organs metastasis and lung plus other organs metastasis in propensity score‐matching (PSM) cohort (A). The PFS rate of lung metastasis (B), other organs metastasis (C) and lung plus other organs metastasis (D) in synchronous and asynchronous treatment groups. CI, confidence interval; HR, hazard ratio.

To further clarify the different types of metastasis on prognosis, patients were subdivided by lung metastasis, other organs metastasis and lung plus other organs metastasis. After propensity matching, significant difference was observed in OS (Figure 4A) and PFS (Figure 5A) among the three types of metastases. In the PSM cohort, the median OS and PFS of patients with lung metastasis were 28.0 and 12.8 months and 16.4 and 8.1 months in synchronous and asynchronous treatment groups, respectively, and there was obvious difference in OS (HR, 0.26; 95% CI, 0.13–0.51; *p* < .001) (Figure 4B) and PFS (HR, 0.33; 95% CI, 0.20–0.55; *p* < .001) (Figure 5B) between the two groups. The median OS and PFS for patient with other organs metastasis were 20.4 and 9.0 months and 17.1 and 7.3 months in synchronous and asynchronous treatment groups, respectively. Significant difference was observed in OS (HR, 0.51; 95% CI, 0.27–0.96; *p* = .036) (Figure 4C) and PFS (HR, 0.56; 95% CI, 0.32–0.98; *p* = .038) (Figure 5C) between the two groups. Similarly, in patients with lung plus other organs metastasis, significant difference was observed in OS (HR, 0.49; 95% CI, 0.25–0.98; *p* = .045) (Figure 4D) and PFS (HR, 0.51; 95% CI, 0.26–0.98; *p* = .042) (Figure 5D) between synchronous and asynchronous treatment groups. The OS and PFS were significantly improved after concomitant use of lenvatinib plus PD‐1 inhibitor among the three groups.

### 3.5 Efficacy evaluation

Efficacy data were evaluated according to RECIST 1.1 evaluation (Table 2). In the 3‐month evaluation, ORR were 19.8% and 35.9%, and the DCR were 73.5% and 81.50% in asynchronous and synchronous treatment groups, respectively. The proportions of PR, SD and PD in the two groups were obviously different (*p* < .028) (Table 2). In the 6‐month evaluation, there was one patient who achieved CR in the synchronous treatment group, and ORR were 17.4% and 34.8%, and the DCR were 43.8% and 56.5% in asynchronous and synchronous treatment groups, respectively. The proportions of CR, PR, SD and PD in the two groups were obviously different (*p* < .027) (Table 2).

**TABLE 2:** Efficacy outcomes in patients with advanced hepatocellular carcinoma beyond oligometastasis in different treatment groups.

Variables	Evaluation	Asynchronous group (*n* = 121)	Synchronous group (*n* = 92)	*p*‐Value
3‐Month evaluation	CR	0	0	.028
	PR	24 (19.8)	33 (35.9)	
	SD	65 (53.7)	42 (45.7)	
	PD	32 (26.4)	17 (18.4)	
6‐Month evaluation	CR	0	1 (1.1)	.027
	PR	21 (17.4)	31 (33.7)	
	SD	32 (26.4)	20 (21.7)	
	PD	68 (56.2)	40 (43.5)	

Abbreviations: CR, complete response; PD, progressive disease; PR, partial response; SD, stable disease.

## 4 Safety

All 213 patients received lenvatinib and PD‐1 inhibitor at different time points. Treatment‐related AEs occurred in most patients and the main AEs are recorded in Table S2. Of all AEs, patients had the highest incidence of decreased appetite, with 50 (41.3%) patients in the asynchronous treatment cohort and 37 (40.2%) patients in the synchronous treatment cohort. No therapy‐related deaths were reported in either group. Patients with grades 1–2 of AEs received suggestive therapy or dosage decrease and experienced relief. For grades 3–4 of AEs, lenvatinib and PD‐1 inhibitor administration was temporarily stopped until the symptoms abated or disappeared. If possible, after recovery, patients resumed PD‐1 inhibitor infusion and a lower dose of lenvatinib.

## 5 DISCUSSION

Recently, the landscape of treatment options for advanced HCC has become significant. Prior to the availability of new options in the last decade, sorafenib was the sole available option for advanced HCC, and there are now new options to treat patients under various conditions.^32^ The Food and Drug Administration (FDA) had approved atezolizumab plus bevacizumab (IMbrave150 trial) and lenvatinib as the first‐line drugs and pembrolizumab and nivolumab as the second‐line drugs for advanced HCC therapy.^33^, ^34^ While lenvatinib and PD‐1 inhibitor demonstrated effectiveness among a portion of patients with advanced HCC, the response to monotherapy remains suboptimal. Clinical evidence indicates that lenvatinib combined with pembrolizumab is effective and promising during the course of treatment of advanced HCC.^23^ The advent of this type of tyrosine kinase inhibitor and immune checkpoint inhibitor ushered in a new chapter that opened for advanced HCC therapies. In this multi‐centre investigation, we compared the synchronous and asynchronous applications of PD‐1 inhibitor in patients with advanced HCC beyond oligometastasis. Data exhibited that synchronous therapy using lenvatinib with PD‐1 inhibitor led to significant survival improvements.

In the well‐designed prospective trials, the administration of lenvatinib and PD‐1 inhibitor was simultaneous.^23^ There are no related reports on the administration of lenvatinib plus PD‐1 inhibitor in patients with advanced HCC beyond oligometastasis. In fact, not all patients received the two drugs at the same time, which involves doctor's recommendations, patient choice and economic affordability. Some patients received lenvatinib firstly, and then combined with PD‐1 inhibitor after an unsatisfactory effect of lenvatinib alone. Thus, we comprehensively compared the prognosis of different synchronous uses of lenvatinib and PD‐1 inhibitors in real‐world applications. Patients were classified into two groups: (1) lenvatinib plus PD‐1 inhibitor were used synchronously (synchronous treatment group); (2) patients received PD‐1 inhibitor after 3 months of lenvatinib treatment prior to TP (asynchronous treatment group). The results showed that lenvatinib with synchronous administration of PD‐1 inhibitor was superior to asynchronous therapy in patients with advanced HCC beyond oligometastasis. After propensity matching, the median OS of synchronous treatment group was 24.6 months, which was 9.8 months longer than asynchronous treatment group (14.8 months). The median PFS of synchronous treatment group was 10.5 months, which was 6.9 months longer than asynchronous treatment group (3.6 months). Compared with asynchronous treatment, synchronous treatment was related to significantly better OS (HR, 0.45; 95% CI, 0.30–0.66; *p* < .001) and PFS (HR, 0.46; 95% CI, 0.33–0.63; *p* < .001) in patients with advanced HCC beyond oligometastasis.

The median OS and PFS of lenvatinib plus pembrolizumab administered simultaneously were 22.0 and 9.3 months in unresectable HCC, respectively.^23^ Our results in synchronous treatment group were 24.6 and 10.5 months, which were comparable to those reported.^23^ Additionally, the 6‐ and 12‐month OS rates were 100% and 71.5% in asynchronous treatment group, which were also higher than the OS rates in atezolizumab plus bevacizumab treatment (84.8% and 67.2%). The main reason for this may be that most patients had a history of local treatment, while patients in lenvatinib–pembrolizumab and atezolizumab–bevacizumab trials had naive unresectable HCC. Despite the more advanced stage of HCC in our report, most patients accepted the TACE, HAIC, ablation or radiotherapy before inclusion in the analysis, and the primary tumour or metastases were controlled to some degree. Another reason was that local treatment induced the tumour necrosis, which stimulated the systemic immune response. Studies have suggested that the lenvatinib boosts the effectiveness of the PD‐1 inhibitor by reversing immunosuppressive effects of vascular endothelial growth factor (VEGF) in the tumour microenvironment, thus enhancing the tumour responsiveness to the combination therapy.^35^, ^36^

The results of the REFLECT study of lenvatinib in primary unresectable HCC showed that the median OS and PFS were 13.6 and 8.9 months, respectively.^19^ In our study, the median OS and PFS in asynchronous treatment group were 14.8 and 6.9 months, respectively. Although the PFS was shorter than that in the REFLECT study, the OS was longer. One reason may be that the patients enrolled in our study were more advanced, and it is acceptable that PFS was shorter. Another reason was that patients benefited more after adding the PD‐1 inhibitor than REFLECT after lenvatinib treatment progression, and there was almost 8 months post‐PFS survival. The FDA has approved the atezolizumab plus bevacizumab as the primary treatment option for advanced HCC since 2020. The PFS and OS are surprising, while most patients cannot afford the expense of atezolizumab plus bevacizumab in China. Our study suggested that HCC patients beyond oligometastasis could obtain obvious clinical benefit from the simultaneous use of lenvatinib and PD‐1 inhibitor treatment, which brings more options and hope to patients.

Several factors can impact the OS of patients with advanced HCC, including the primary tumour characteristics, underlying liver disease, patient immune and inflammatory status and the chosen treatment method. In our study, multivariate analysis revealed that treatment patterns, metastasis location, AST level and PVTT were identified as significant factors impacting OS in patients with advanced HCC beyond oligometastasis. Earlier research has reported that PVTT is a crucial factor related to poorer OS,^37^, ^38^ and extrahepatic metastases have also proved as a poor prognostic factor in advanced HCC.^10^ Lung is the most common extrahepatic metastatic site for patients with advanced HCC (followed by lymph nodes, bones and adrenal glands), almost accounting 20%–40% of HCC metastases.^10^, ^39^ However, there are few reports on the prognosis of different extrahepatic metastases. In the present study, we classified metastases into three types: lung metastasis, other organs metastasis and lung plus other organs metastasis, and further analysed the prognosis of different treatments (asynchronous treatment and synchronous treatment) with different types of metastases. The results showed that patients who received lenvatinib plus PD‐1 inhibitor synchronously had remarkably better OS and PFS than asynchronous treatment in three types of metastasis groups. Additionally, we observed that patients with lung metastasis had a remarkably better prognosis compared to those with other organs metastasis or lung plus other organs metastasis. These observations suggest that adequate assessment of the disease and selection of appropriate treatment options are important for improving patient outcomes.

There are several limitations that should be illustrated. Firstly, this study is retrospective and lacked randomisation, which may have led to a biased selection. However, we attempt to mitigate this limitation through the PSM. Secondly, this study represents an actual utilisation of lenvatinib and PD‐1 inhibitor, and the influence of physician and patient discretion in patient enrollment and medication selection cannot be fully eliminated. Thirdly, while data from multiple centres were included, the sample size of patients analysed may be limited, potentially impacting the results. Further validation of our study's results is necessary by extensive multi‐centre investigation and randomised trials.

## 6 CONCLUSIONS

In conclusion, this study demonstrates that synchronous use of lenvatinib and PD‐1 inhibitors results in clinically significant improvements in advanced HCC beyond oligometastasis. Treatment of lenvatinib with PD‐1 inhibitors simultaneously may be a promising strategy for their complementary effect in primary unresectable HCC patients with multi‐metastases.

## CONFLICT OF INTEREST STATEMENT

The authors declare no conflicts of interest.

## Supporting information

Supporting‐Information
