# Supplementary material for: Effectiveness of lenvatinib plus immune checkpoint inhibitors in primary advanced hepatocellular carcinoma beyond oligometastasis
Source: Clin Transl Med. 2023 Feb 28;13(3):e1214. doi: 10.1002/ctm2.1214 (PMC9975463; doi:10.1002/ctm2.1214)
Supplement: Supplementary file 1 — Supporting‐Information [file CTM2-13-e1214-s001.docx]

**Effectiveness of Lenvatinib plus Immune Checkpoint Inhibitors in Primary Advanced Hepatocellular Carcinoma beyond Oligometastasis**

Xiao-Hui Wang^1^, Chang-Jun Liu^1^, Hao-Quan Wen^1^, Xiao-Hui Duan^1^, Yu-Qing Jiao^2^, Yu-Jiang Liu^3^, Min-Shan Chen^4^, Kang-Shun Zhu^2#^, Xian-Hai Mao^1#^, Qun-Fang Zhou^3#^

^1^Department of Hepatobiliary Surgery, Hunan Provincial People's Hospital (The First Affiliated Hospital of Hunan Normal University) Changsha, 410005, Hunan province, China.

^2^Department of Minimally Invasive Interventional Radiology, and Department of Radiology, the Second Affiliated Hospital of Guangzhou Medical University, Guangzhou, 510260, China.

^3^Department of Interventional Ultrasound, Chinese PLA General Hospital, 28 Fuxing Road, Beijing 100853, China.

^4^Department of Liver Surgery, Sun Yat-Sen University Cancer Center, Guangzhou, Guangdong, 510060, P. R. China.

**^#^Correspondence authors:**

Xian-Hai Mao，Department of Hepatobiliary Surgery, Hunan Provincial People's Hospital (The First Affiliated Hospital of Hunan Normal University) Changsha, 410005, Hunan province, China. Email: [[maoxh2022@hunnu.edu.cn](mailto:mxhaiszy@yahoo.com)](mailto:maoxh2022@hunnu.edu.cn); Tel: +86-0731-83928052;

Kang-Shun Zhu，Department of Minimally Invasive Interventional Radiology, and Department of Radiology, the Second Affiliated Hospital of Guangzhou Medical University, Guangzhou, 510260, China. Email: [[zhksh010@163.com](mailto:zhksh010@163.com)](mailto:zhukangshun@gzhmu.edu.cn); Tel: +86-020-34152299;

Qun-Fang Zhou，Department of Interventional Ultrasound, Chinese PLA General Hospital, 28 Fuxing Road, Beijing 100853, China. Email: [zhouqun988509@163.com](mailto:zhouqun988509@163.com); Tel: +86-010-68182255;

**Supplementary Figure 1.** Distribution of the propensity score after matching.

**Supplementary Figure 2.** Standardized differences in mean or proportion of variables before and after propensity score matching.

**Supplementary table 1.** Univariate analysis of prognostic factors for progression-free survival (PFS) and overall survival (OS) in patients with advanced hepatocellular carcinoma (HCC) beyond oligometastasis.

**Supplementary table 2.** Most common treatment-related adverse events in patients receiving Lenvatinib and PD-1 inhibitor in two groups.

**Supplementary Figure 1.** Distribution of the propensity score after matching.


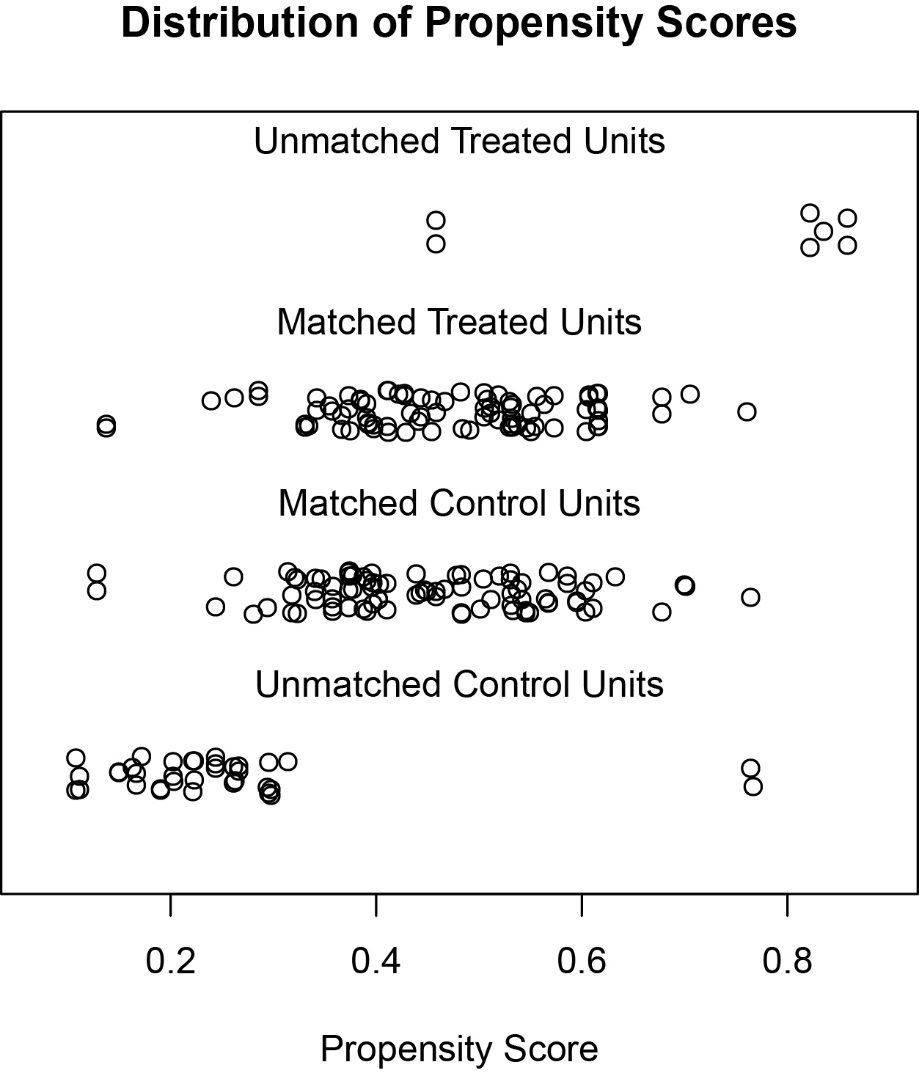


**Supplementary Figure 2.** Standardized differences in mean or proportion of variables before and after propensity score matching


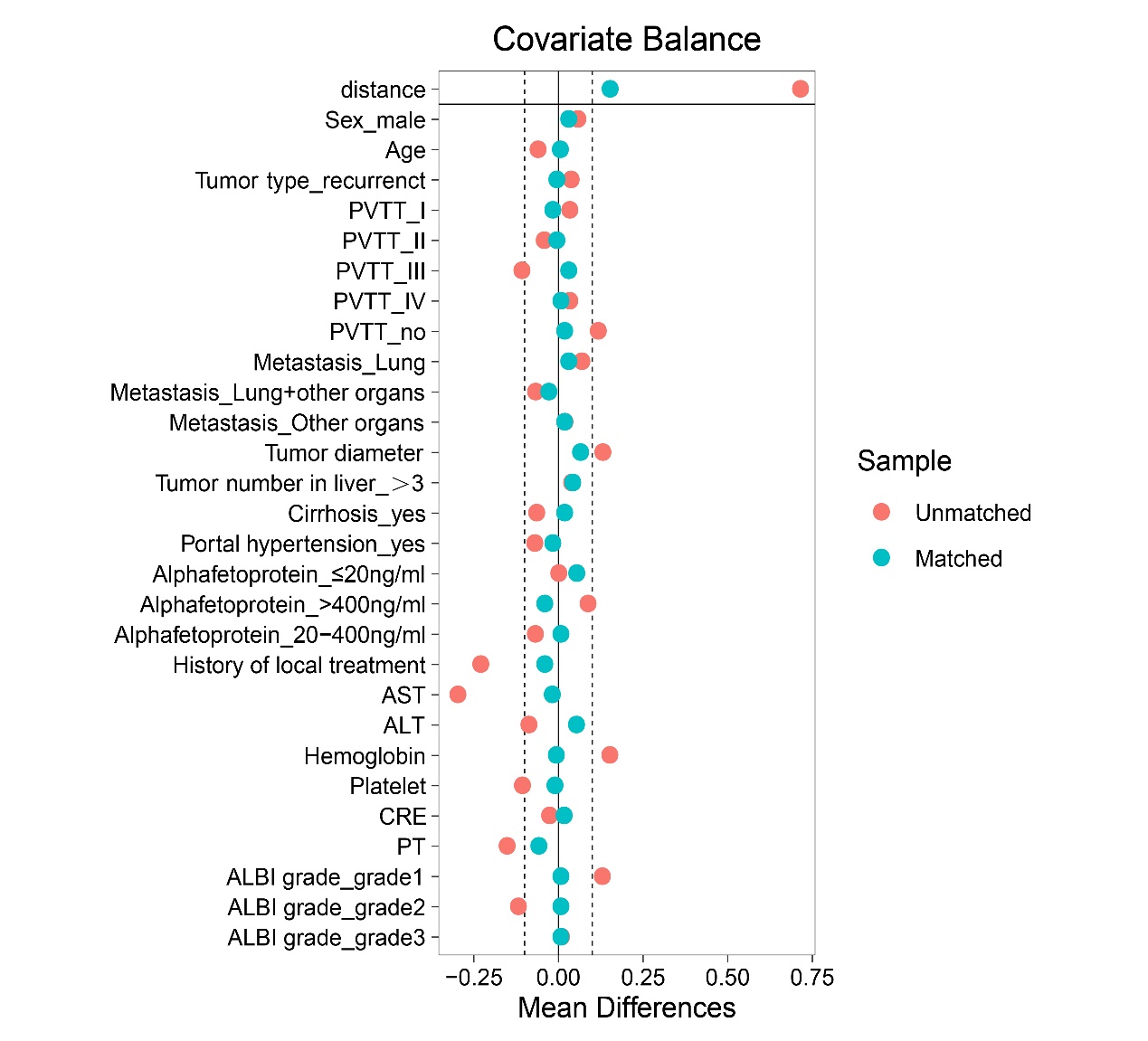


**Supplementary table 1**. Univariate analysis of prognostic factors for progression-free survival (PFS) and overall survival (OS) in patients with advanced hepatocellular carcinoma (HCC) beyond oligometastasis

| **Characteristics** | **Comparison** | **Progression-free survival** | | **Overall survival** | |
| --- | --- | --- | --- | --- | --- |
|  |  | **HR (95% CI)** | ***P value*** | **HR (95% CI)** | ***P value*** |
| **Sex** | male vs. female | 0.87 (0.55-1.39) | 0.569 | 1.09 (0.62-1.91) | 0.774 |
| **Age,** years | <60 vs. ≥60 | 0.70 (0.46-1.07) | 0.103 | 0.66 (0.37-1.17) | 0.155 |
| **HBsAg** | negative vs. positive | 1.06 (0.72-1.55) | 0.778 | 0.76 (0.48-1.21) | 0.244 |
| **Cirrhosis** | No vs. yes | 0.81 (0.59-1.11) | 0.185 | 1.10 (0.74-1.62) | 0.645 |
| **Portal hypertension** | No vs. yes | 1.33 (1.02-1.77) | 0.041 | 1.52 (1.08-2.15) | 0.017 |
| **Local treatment history** | No vs. yes | 1.21 (0.87-1.69) | 0.262 | 1.47 (0.94-2.30) | 0.093 |
| **PVTT** | No  I  II  III  Ⅳ | Reference  1.52 (0.89-3.11)  1.61 (0.68-3.25)  1.73 (1.06-3.06)  1.75 (1.05-3.22) | 0.105  0.386  0.012  0.008 | Reference  1.70 (1.22-3.54)  1.86 (1.28-3.75)  2.05 (1.26-4.09)  2.28 (1.29-4.71) | 0.012  0.001  <0.001  <0.001 |
| **Metastasis** | Lung  Other organs  Lung+ other organs | Reference  1.30 (0.91-1.85)  1.89 (1.28-2.80) | 0.153  0.001 | Reference  1.43 (0.90-2.27)  3.11 (1.90-5.10) | 0.126  <0.001 |
| **AFP,** ng/mL | <20  ≥20, <400  ≥400 | Reference  1.06 (1.01-1.62)  1.33 (1.03-1.89) | 0.038  0.012 | Reference  0.94 (0.52-1.70)  1.26 (0.75-2.13) | 0.846  0.379 |
| **Treatment Patterns** | Synchronous  Asynchronous | Reference  2.20 (1.59-3.03) | <0.001 | Reference  2.24 (1.50-3.32) | <0.001 |
| **ALBI grade** | Grade 1  Grade 2  Grade 3 | Reference   - 1. (0.70-1.31)   0.96 (0.42-2.22) | 0.798  0.930 | Reference  1.17 (0.79-1.74)  1.48 (0.53-4.13) | 0.438  0.454 |
| **Tumor number in liver** | ≤3 vs. >3 | 1.26 (0.90-1.77) | 0.180 | 1.98 (1.23-3.19) | 0.005 |
| **ALT, U/L** | ≤40 vs. >40 | 1.07 (0.79-1.44) | 0.682 | 1.28 (0.87-1.89) | 0.213 |
| **AST, U/L** | ≤40 vs. >40 | 1.35 (1.01-1.88) | 0.047 | 1.94 (1.23-3.06) | 0.005 |

ALT, alanine aminotransferase; AST, aspartate aminotransferase; AFP, alpha-fetoprotein; ALBI, albumin-bilirubin; HBsAg, hepatitis B surface antigen; PVTT, portal vein tumor thrombus;

**Supplementary table 2**. Most common treatment-related adverse events in patients receiving Lenvatinib and PD-1 inhibitor in two groups.

| **Adverse events** | **Asynchronous**  **Group**  (n=121) | **Synchronous**  **Group**  (n=92) | ***P* value** |
| --- | --- | --- | --- |
| **Hypertension**  Grade 1–2  Grade 3-4 | 41 (33.9)  8 (6.6) | 34 (36.9)  6 (6.5) | 0.896 |
| **Fatigue**  Grade 1–2  Grade 3-4 | 39 (32.2)  3 (2.5) | 27 (29.3)  2 (2.2) | 0.887 |
| **Rash or desquamation**  Grade 1–2  Grade 3-4 | 20 (16.5)  2 (1.6) | 16 (17.4)  1 (1.1) | 0.931 |
| **Diarrhea**  Grade 1–2  Grade 3-4 | 35 (28.9)  1 (0.8) | 25 (27.2)  0 (0.0) | 0.649 |
| **Decreased appetite**  Grade 1–2  Grade 3-4 | 50 (41.3)  0 (0.0) | 37 (40.2)  0 (0.0) | 0.871 |
| **Increased ALT/AST**  Grade 1–2  Grade 3-4 | 19 (15.7)  0 (0.0) | 14 (15.2)  1 (1.1) | 0.515 |
| **Proteinuria**  Grade 1–2  Grade 3-4 | 20 (16.5)  3 (2.5) | 19 (20.7)  3 (3.3) | 0.685 |
| **Hypothyroidism**  Grade 1–2  Grade 3-4 | 29 (23.9)  0 (0.0) | 19 (20.7)  0 (0.0) | 0.566 |
| **Weight decreased**  Grade 1–2  Grade 3-4 | 11 (9.1)  0 (0.0) | 8 (8.7)  0 (0.0) | 0.767 |

ALT, alanine aminotransferase; AST, aspartate aminotransferase;
